# Poisoning by Chloral—Antidote

**Published:** 1876-04

**Authors:** 


Poisoning by Chloral—Strychnia as an Antidote—Recovery.—Dr. Levenstein reports the following case (*Centralbl. f. Med. Wissen.*) A man who had taken about six drachms of chloral was found half an hour afterwards in deep sleep, but without alarming symptoms. A little later, however, tumultuous action of the heart, with difficult and interrupted respiration, set in; and later still the action of the heart became greatly enfeebled, so that, even in the carotids, scarcely any pulse was discernable. The pupils were greatly contracted, and the temperature sank to 91.2°.

Artificial respiration, by passive movement and faradization, proving of no avail, one-twenty-second of a grain of strychnia was administered hypodermically. Convulsive movements of the muscles almost immediately ensued, and soon after trismus; the cardiac movement again became perceptible, the pupils dilated, and the temperature rose to 92 deg. Shortly after, the former threatening symptoms appearing once more, a second injection of strychnia, to the amount of one-thirty-second of a grain, was administered. The effect of this was similar to that of the first, the temperature rising to normal; but artificial respiration had to be kept up for eight hours. Trismus and tetanic spasm remained for fourteen hours after the second injection; but at the end of the thirty-two hours the patient awoke feeling fresh and well. No gastritis followed the ingestion of the chloral, probably because the patient's stomach contained food at the time.—*Med. Brief.*

